# Paradoxical Reasoning: An fMRI Study

**DOI:** 10.3389/fpsyg.2022.850491

**Published:** 2022-05-02

**Authors:** Antigoni Belekou, Charalabos Papageorgiou, Efstratios Karavasilis, Eleftheria Tsaltas, Nikolaos Kelekis, Christoph Klein, Nikolaos Smyrnis

**Affiliations:** ^1^Laboratory of Cognitive Neuroscience and Sensorimotor Control, University Mental Health, Neurosciences and Precision Medicine Research Institute “COSTAS STEFANIS”, Athens, Greece; ^2^First Department of Psychiatry, Eginition Hospital, Medical School, National and Kapodistrian University of Athens, Athens, Greece; ^3^Second Department of Radiology, General University Hospital “ATTIKON”, Medical School, National and Kapodistrian University of Athens, Athens, Greece; ^4^Second Department of Psychiatry, General University Hospital “ATTIKON”, Medical School, National and Kapodistrian University of Athens, Athens, Greece; ^5^Department of Child and Adolescent Psychiatry, Medical Faculty, University of Freiburg, Freiburg, Germany; ^6^Department of Child and Adolescent Psychiatry, Medical Faculty, University of Cologne, Cologne, Germany

**Keywords:** paradoxical syllogism, deductive reasoning, fMRI, fronto-parietal brain activation patterns, fronto-temporal brain activation patterns

## Abstract

Paradoxes are a special form of reasoning leading to absurd inferences in contrast to logical reasoning that is used to reach valid conclusions. A functional MRI (fMRI) study was conducted to investigate the neural substrates of paradoxical and deductive reasoning. Twenty-four healthy participants were scanned using fMRI, while they engaged in reasoning tasks based on arguments, which were either Zeno’s like paradoxes (paradoxical reasoning) or Aristotelian arguments (deductive reasoning). Clusters of significant activation for paradoxical reasoning were located in bilateral inferior frontal and middle temporal gyrus. Clusters of significant activation for deductive reasoning were located in bilateral superior and inferior parietal lobe, precuneus, and inferior frontal gyrus. These results confirmed that different brain activation patterns are engaged for paradoxical vs. deductive reasoning providing a basis for future studies on human physiological as well as pathological reasoning.

## Introduction

The unique reasoning ability of the human brain has been the focus of studies within philosophy and psychology for over 50 years (Monti and Osherson, 2011). The process of reasoning is one of the advanced human intellectual abilities and is therefore a hallmark of higher cognition (Rodriguez-Moreno and Hirsch, 2009; Papaodysseus et al., 2016). Aristotle was the first philosopher who attempted to analyze logical reasoning by means of deduction (Aristotle, *Prior Analytics*; Cooke and Tredennick, 1938). Deductive reasoning is a logical process of evaluating arguments. Namely, all arguments start with a set of sentences (premises) which provide some grounds for accepting the conclusion (Aristotle, *Posterior Analytics*; Tredennick and Forster, 1960). Deductive arguments are evaluated in terms of their validity and soundness. A valid argument implies that its premises provide absolute grounds for accepting the conclusion (ignoring their actual truth values), whereas an argument is invalid when the conclusion has not come up with logical certainty by the premises (even if the premises and the conclusion are true). An argument is sound if it is both valid and all of its premises are actually true (Aristotle, *Posterior Analytics*; Tredennick and Forster, 1960). Α typical example of deductive reasoning is the following: “All men are mortal. All Athenians are men. All Athenians are mortal.” The above premises provide the claim with absolute certainty that “All Athenians are mortal” (Barnes, 1995). Deduction consists of a process of drawing necessary conclusions from previous items of knowledge and reasoners beliefs about the world and intuition could affect their judgement and performance in typical reasoning tasks (De Neys, 2006). Recent studies have proposed that the reasoners tend to accept the believable conclusions as valid, ignoring the logical validity (belief bias effect; Goel and Dolan, 2003; Morley et al., 2004).

A number of neuroimaging studies have examined the neural substrate of deductive reasoning in relation with other forms of reasoning, such as relational, conditional, inductive, probabilistic reasoning or spatial, belief-laden, content-based/no-content-based and emotional reasoning. Psychological theories have introduced specific models regarding the nature of the mental representations that support deductive syllogisms (Prado et al., 2011). Two hypotheses were formed based on these studies, namely that deductive reasoning is a visuo-spatial process or a linguistic process that relies on rule-based mechanisms (Plutarch, 1916; Goel et al., 2000; Knauff et al., 2002; Goel and Dolan, 2004; Kroger et al., 2008; Brunetti et al., 2014). The advocates of Mental Model Theory propose that deductive reasoning is a visuo-spatial process, where a spatial representation of the problem premises is constructed, manipulated, and evaluated (Johnson-Laird, 2001, 2010). Hence, if reasoning is spatial in nature, then the brain regions that should be activated during deduction could be also involved in visuospatial processing, such as right hemisphere parieto-occipital areas (Goel, 2007; Prado et al., 2011). On the other hand, the exponents of Formal Rule Approach (FRA) suggest that reasoning relies on rule-based mechanisms and that deduction is evaluated by a set of linguistic rules sensitive to the logical form of the argument (Braine, and O’Brien, 1998). So, if reasoning is a linguistic process, then it should be supported by left hemisphere language areas (Goel, 2007; Rodriguez-Moreno and Hirsch, 2009).

Another model for reasoning is the theory of dual-process reasoning (Evans, 2002, 2003; Goel and Dolan, 2003; De Neys, 2006). According to this model, there are two reasoning systems subserved by distinct neurobiological substrates. The first system (heuristic) is a rapid, parallel, automatic process, which activates fronto-temporal areas and is responsible for judgements based on whether the conclusion of an argument is a true statement (belief bias effect). The second system (analytic) is a slow, sequential process, which activates brain regions in parieto-occipital areas and working memory associated areas. Judging the logical validity of deductive syllogisms is thought to depend on the activation of this second system, which entails “effortful hypothetical thinking” (Stephens et al., 2020).

Conversely to Aristotle’ s reasoning, about 2,500 years ago, Zeno of Elea, student of Parmenides, introduced the paradoxical syllogism. Zeno introduced the paradoxes as a method of indirect proof (“reduction ad absurdum”). Namely, this method requires temporarily assuming some thesis, which the orator is in fact opposed to, and then attempting to deduce an absurd conclusion or a contradiction and by that means undermining the original supposition (Plutarch, 1916; Papageorgiou et al., 2016). A paradox is *a statement or a proposition that despite apparently sound reasoning from acceptable-valid premises, leads to a conclusion that appears self-contradictory or logically unacceptable -from the common sense-* (Oxford Wordpower Dictionary, 1993). The paradoxical reasoning array comprises statements of the following type: “Achilles and the tortoise decide to race. The tortoise gains a head start, because it runs slowly. Therefore, the tortoise will always be ahead of Achilles.” The Achilles paradox proves that the slow-moving tortoise will never be passed by the fleet-footed Achilles. Namely, Achilles should first arrive at the point that the tortoise started in order to get ahead of the tortoise. However, by the time that Achilles will have reached to that point, the tortoise will have moved to another point and so on. Consequently, the initial head start that was given to the tortoise was a determining factor to its victory. Another example of a paradoxical statement is the following: “Epimenides claims that all Cretans are liars.” Epimenides was Cretan. Consequently, if we assume that his statement is true, it means that Epimenides was a liar. Therefore, Epimenides statement about Cretans was a lie. Hence, Cretans do not lie, but tell the truth. Since Epimenides was Cretan, his words were true and so on. Many scientific fields as Philosophy and Mathematics have been intrigued by Zeno’s paradoxes, and this way of thinking was characterized as a form of cognitive illusion (Papaodysseus et al., 2016). Indeed, it has been remarked that such cognitive illusions breach the norms of rational thought only in the context of philosophical speculation (Atmanspacher et al., 2004; Papageorgiou et al., 2016).

The cognitive processes and the functional neuroanatomy underlying paradoxical reasoning have not been investigated. An interesting question that arises is whether the same brain system or different ones are activated when humans are faced with paradoxical reasoning (such as the case of Zeno’s paradoxes) or logical reasoning (as the case of deductive reasoning). This question was addressed in a previous event potential (ERP) study (Papageorgiou et al., 2016) where it was investigated whether valid deduction and paradoxical syllogism would elicit similar or different patterns of electrophysiological activity reflected in cognitive ERPs, such as P300. The results suggested that the patterns of activation for logical and paradoxical reasoning were different. Deductive reasoning elicited activity in parietal-occipital areas, whereas paradoxical reasoning elicited activity in frontal/orbitofrontal brain areas (Papageorgiou et al., 2016). Paradoxes are also associated with irony, contradiction, inconsistency, and oxymoron (Smith et al., 2017) suggesting that they might be a non-literal form of language. Rapp et al. (2012) proposed the existence of a fronto-temporal network that is entailed in the comprehension of non-literal or figurative language. Thus one could hypothesize that paradoxes would activate this fronto-temporal network.

Most studies, until now, have investigated the neural bases of the so-called logical syllogism. The current study aims to specifically investigate the functional neuroanatomy supporting reasoning in a non-typical logical form, as paradoxes, using fMRI and furthermore define common or different activation patterns related to deductive vs. paradoxical reasoning. To address these questions, we compared activation patterns while participants attempted to come to a logical conclusion regarding the correctness of paradoxes or valid syllogisms. There was also, a baseline condition, in which participants were required to evaluate the words ‘spelling of similar arguments. This study aimed to take advantage of the high spatial resolution of fMRI as well as its ability to capture activation patterns in the whole brain. Our results suggest that different brain activation patterns are engaged for paradoxical vs. deductive reasoning.

## Materials and Methods

### Participants

Twenty-four right-handed young volunteers (12 men and 12 women), with a mean age of 24.67 years (*SD* = 3.3) and mean education level of 16 years (*SD* = 0.94) participated in this study. Four participants were excluded from the analysis (two due to noise in MRI images, two due to missing data). Participants spoke Greek as their native language. They all had normal or corrected-to-normal vision and were screened for the absence of neurological or psychiatric disorders. All participants gave signed informed consent, after being thoroughly informed about the procedure.

### Procedures

Verbal instructions, followed by two examples of valid, two of invalid, and two of paradoxical statements, were given to each participant as training in order to ensure that the participant had totally comprehended the task. The participant was instructed to ignore the actual truth value of the conclusion regarding each syllogism and emphasize on evaluating the validity of the arguments (whether the conclusion follows logically from the premises). It should be noted here that all participants had experience in similar tests of logical reasoning that are taught in the Greek high school education.

During the fMRI scanning procedure, participants laid in the MR-scanner with their head secured by foam rubber to minimize movement artefacts. The participants were shielded from noise by earplugs and headphones. Stimuli were presented using a projector outside the magnet room projecting onto a screen in the magnet room that was viewed by the participant *via* a mirror mounted on the head coil. The sentences were projected with black letters on a white background. E-prime software (Psychology Software Tools, Pittsburgh, PA, United States) running on a personal computer was used to generate visual stimuli and control experimental parameters.

### Stimuli

The task was designed to compare two mental functions: processing of syllogisms characterized as paradoxical to processing of valid reasoning. Fifty deductive reasoning syllogisms (25 valid, 25 invalid), 25 paradoxes and 25 baseline trials (control condition) were generated. All valid deductive syllogisms resulted in true conclusions while 20 of the 25 invalid syllogisms resulted in non-true conclusions and five resulted in true conclusions. Stimuli from all conditions were presented pseudo-randomly, in an event-related design. The valid and paradoxical statements that we used are translated and available in a supplementary file submitted to the journal. The experiment was separated in three blocks of 12 min. The order of the trials at each block was counterbalanced. The onset of a deductive syllogism was signaled by a “+” sign, the onset of a paradoxical syllogism by an “*” sign, and the onset of a control trial by an “X” sign (Table 1). All triplets of sentences for each trial appeared on the screen simultaneously and remained visible for 16 s.

**Table 1:** Stimuli presentation.

Deductive reasoning	Paradoxical reasoning
+	*
All men are mortal. All Greeks are men. All Greeks are mortal.	Several documents include pages which have the phrase: ‘This page was intentionally left blank’. So, that page is blank.
Right or Wrong?	Right or Wrong?

In the deduction reasoning trials, participants were required to determine whether the given conclusion followed logically from the premises, thus responding that it was right or wrong (i.e., valid or invalid syllogisms). In the paradoxical reasoning trials, participants were required to determine and respond whether the given conclusion was right or wrong. Finally, in control trials, participants were required to examine and respond whether the words’ spelling of the arguments was correct or wrong. Participants were verbally instructed to read carefully each statement, followed by the question “Right or Wrong?” and respond whether the statement was right or wrong, as quickly as possible. Correct and erroneous responses were recorded as key presses of two buttons on a MR compatible keypad.

### fMRI Acquisition Protocol

Functional MRI was performed using a 3.0 T Achieva TX, Philips manufactured scanner equipped with an eight channel head coil. The brain imaging protocol consisted of axial T2-FLAIR, sagittal high resolution 3DT1-TFE and axial T2* EPI BOLD sequences. The used parameters in BOLD sequence were TR = 2,000 ms, TE = 3 0 ms, flip angle = 90^o^, field of view = 256 × 256, acquisition and reconstructed voxel size 3 mm × 3 mm × 3 mm, and sensitivity encoding reduction factor of two. The BOLD sequence was repeated three times with scanning time 12 min for each scan. The scanner was synchronized with the presentation of all trials in each session. The acquisition protocol duration was approximately 45 min. All data underwent visual quality control before further post-processing.

### Data Analysis

Behavioral data analysis was conducted using IBM SPSS software (version 23). For each trial the type of response was recorded (correct/wrong) as well as the Reaction Time for the button press. Percentage of correct responses was calculated for each one of the valid, invalid, and control trials, for each participant. The percentage of correct responses was not measured for the paradoxes since by definition there is no correct response in this case. Indeed, the percentage of correct answers for paradoxes was close to 50% for each participant as expected. The percentage of correct responses for each trial type was entered in a one-way ANOVA with trial type as the within subject factor. The mean response time was also measured for each trial type (valid, invalid, paradox, and control) for each participant. Then, a one-way ANOVA was performed with trial type as the within-subject factor. *Post hoc* comparisons were used to test for differences among the four trial types using the Tuckey Honest significance test.

Analysis of fMRI data was conducted using Statistical Parametric Mapping, version 12 (SPM12; Wellcome Department of Cognitive Neurology, London). Functional image volumes were spatially realigned to the mean image to remove movement artifacts. Slice timing correction was also performed to correct for the slice acquisition during the fMRI scanning. Each participant’s T1 weighted structural images were then co-registered to the mean functional image of each scan, and the spatial normalization parameters from gray and white matter segmentation of anatomical images were used to normalize the realigned functional volumes to the Montreal Neurological Institute (MNI) space. The normalized volumes were then smoothed using an 8 mm full width half maximum Gaussian kernel. Canonical hemodynamic response function (HRF) of SPM12 standard was used to create the fMRI model. At the first level analysis, general linear model (GLM) was used to create *t*-statistical parametric maps for each subject and each session.

For each subject, *t*-test contrasts for the “valid arguments,” “invalid arguments,” and “paradoxes” vs. baseline (control condition) were calculated. These contrasts were calculated at the second level analysis to make inferences at the group level. Moreover, interaction analysis like the following: (valid reasoning—baseline) vs. (paradoxical reasoning—baseline), (invalid reasoning—baseline) vs. (paradoxical reasoning—baseline), (valid reasoning—baseline) vs. (invalid reasoning—baseline), and vice versa was performed. The results of the second-level analysis were corrected using Family Wise Error (FWE) to achieve corrected *p* values (*p* < 0.05). Age and gender were used as nuisance covariates.

The complete data set was transformed into Tailarach space (Talairach and Tournoux, 1988), after correction for differences between the MNI and Tailarach coordinate systems by means of a nonlinear transformation.

## Results

### Behavioral Results

The group mean response accuracy was 84.50% (*SD* = 0.08) for the valid reasoning trials, 84.05% (*SD* = 0.10) for the invalid reasoning trials, and 85.1% (*SD* = 0.07) for the control condition. There was no significant difference in response accuracy among the three conditions [*F* (2, 57) = 0.07, *p* = 0.933]. The group mean response time (RΤ) was 8.7 s (*SD*: 1.21) for the valid reasoning trials, 8.63 s (*SD*: 1.17) for the invalid reasoning trials, 9.77 s (*SD*: 1.15) for the paradoxical reasoning trials, and 7.97 (*SD*: 1.34) for the control condition. There was a significant effect of trial type in RT [*F* (2, 57) = 7.825, *p* < 0.001]. Specifically, *post hoc* comparisons confirmed that the mean RT for valid (Tukey *post hoc* test *p* = 0.029) and invalid reasoning (Tukey *post hoc* test *p* = 0.017), as well as for the control condition (Tukey *post hoc* test *p* < 0.001), was significantly smaller than paradoxical reasoning, while all other comparisons did not reach significance.

### Imaging Results

**Figure 1:**
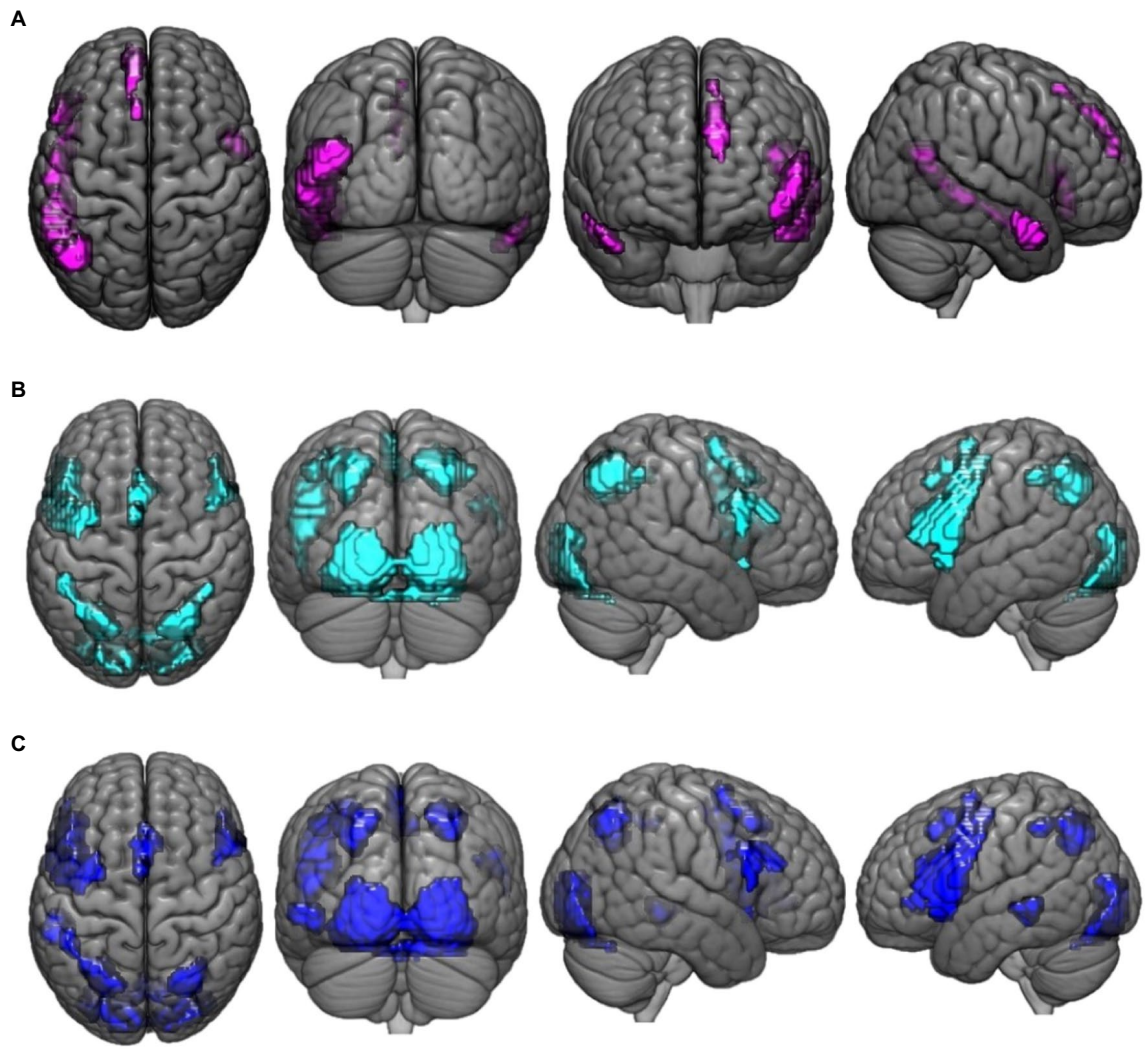
This figure presents the neuroimaging results of the comparison of reasoning trials to the control task: **(A)** activation maps of areas that were significantly more activated during paradoxical reasoning compared to the control task; **(B)** activation maps of areas that were significantly more activated during deductive reasoning for valid syllogisms compared to the control task; and **(C)** activation maps of areas that were significantly more activated during deductive reasoning for invalid syllogisms compared to the control task.

Regions that demonstrated greater activation for paradoxical reasoning compared to control trials are shown in violet in Figure 1A. A number of frontal, temporal, and occipital cortex regions were activated, including bilateral middle temporal gyrus (BA 21, 19), left inferior frontal gyrus (BA 45), superior frontal gyrus (BA 9), and medial frontal gyrus (BA 8).

Regions that demonstrated greater activation for valid reasoning compared to control trials are shown in cyan in Figure 1B. A number of frontal, parietal, and occipital cortex regions were activated including bilateral parietal lobe (precuneus, BA 7), left inferior frontal gyrus (BA 44), lingual gyrus (BA 17), precentral gyrus (BA 6), right inferior frontal gyrus (BA 9), inferior occipital gyrus (BA 17), and cingulate gyrus (BA 32) were more active for valid reasoning vs. control.

Regions that demonstrated greater activation for the invalid reasoning compared to control trials are shown in blue in Figure 1C. Similar areas were more activated for invalid reasoning compared to control trials as were observed for the contrast of valid reasoning vs. control such as bilateral parietal lobe (precuneus, BA 7), left inferior frontal gyrus (BA 44), right inferior occipital gyrus (BA 17), cingulate gyrus (BA 32), inferior frontal gyrus (BA 9), and left middle temporal gyrus (BA 22).

**Figure 2:**
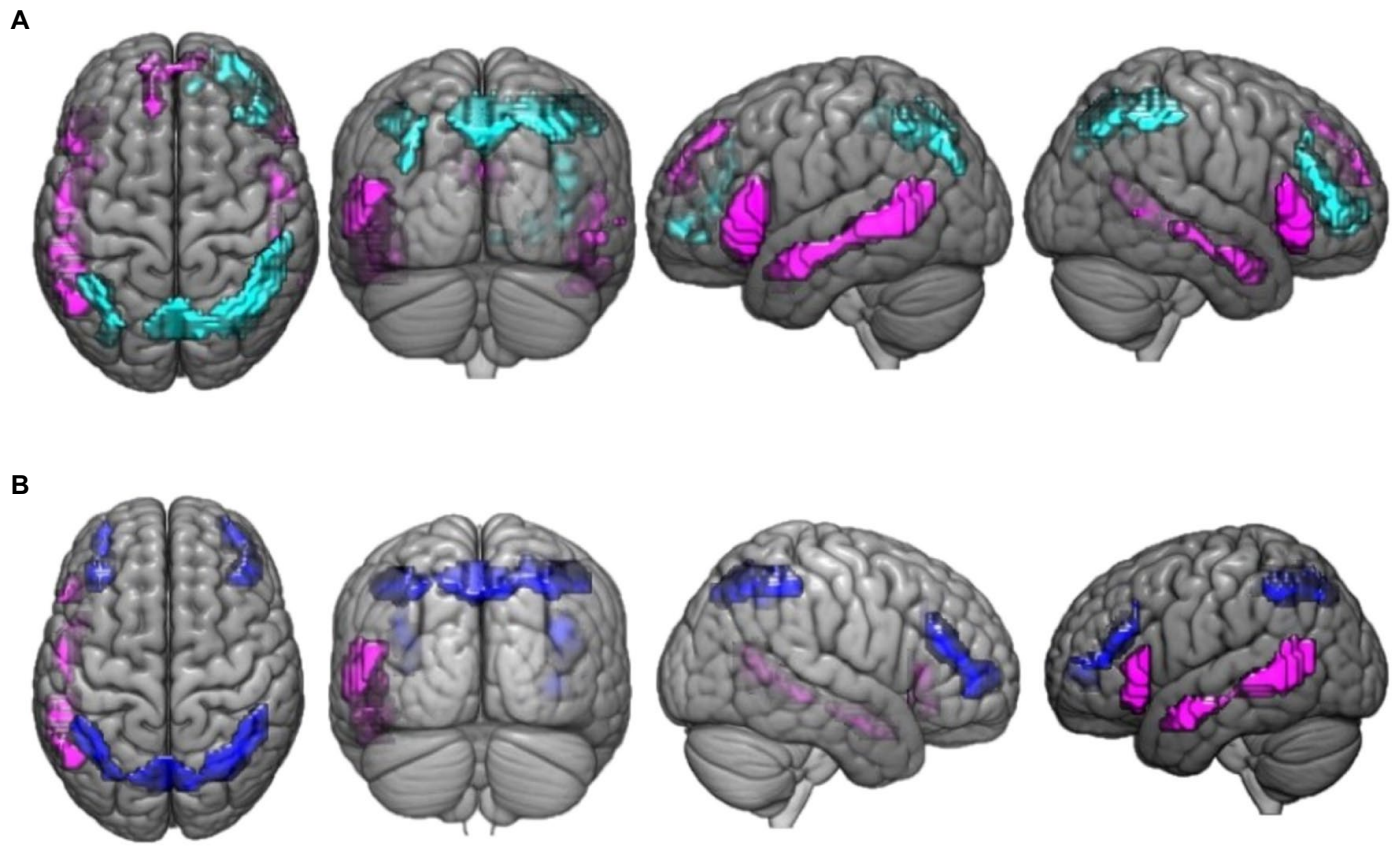
This figure presents the neuroimaging results of the comparison between deductive reasoning and paradoxical reasoning trials: **(A)** activation maps of areas that were differentially activated between deductive reasoning for valid syllogisms and paradoxical reasoning trials. Cyan color marks the areas that were more active for deductive valid reasoning compared to paradoxical reasoning while violet marks the areas that were more active for paradoxical reasoning compared to deductive valid reasoning. **(B)** Activation maps of areas that were differentially activated between deductive reasoning for invalid syllogisms and paradoxical reasoning trials. Blue color marks the areas that were more active for deductive invalid reasoning compared to paradoxical reasoning while violet marks the areas that were more active for paradoxical reasoning compared to deductive invalid reasoning.

The activation differences between valid reasoning and paradoxical reasoning trials are shown in Figure 2A. Valid reasoning preferentially activated bilateral inferior parietal lobe (BA 40), superior parietal lobe (precuneus, BA 7, 19) and right middle frontal gyrus (BA 46, 10). Paradoxical reasoning preferentially activated bilateral middle temporal gyrus (BA 21, 37), inferior frontal gyrus (BA 45, 47), and left medial frontal gyrus (BA 8).

The activation differences between invalid reasoning and paradoxical reasoning trials are shown in Figure 2B. The results reveal bilateral activation clusters associated with deductive (invalid) reasoning. Invalid reasoning preferentially activated bilateral inferior parietal lobe (BA 40), left parietal lobe (precuneus, BA 7), and bilateral middle frontal gyrus (BA 46, 10). Paradoxical reasoning preferentially activated left middle temporal gyrus (BA 21, 37) and inferior frontal gyrus (BA 45).

We also examined whether there were differences in activation for valid vs. invalid reasoning trials. We found no significant differences in activation patterns between these subtypes of reasoning. Finally, we examined whether there were activation differences between “yes/no” responses of the paradoxes. However, no significant differences in activation patterns were found.

## Discussion

This fMRI study aims to provide a different option of the way that human mind functions and investigate the different information processing operations in human mind. In order to address this issue, we designed an evaluation task, where participants compared two mental functions: processing of syllogisms characterized as paradoxical to processing of valid reasoning. Firstly, we compared activation patterns between paradoxical reasoning and a control condition, as well as deductive (valid or invalid) reasoning and a control condition. Then, we compared activation patterns between paradoxical reasoning and deductive reasoning (valid or invalid). Our results confirmed different brain activation patterns for paradoxical vs. deductive reasoning whereas the first activated fronto-temporal regions and the second activated fronto-parietal ones. These findings suggest that reasoning problems engage two distinct systems, which depend on the presence or absence of semantic content (Goel et al., 2000).

### Behavioral Data

There was no difference in accuracy (error rate) or response time between valid and invalid arguments in our study. Although, we have specifically instructed participants to concentrate on the logical validity of each argument and test whether the premises provide absolute grounds for accepting the conclusion there could still be a belief bias effect in deductive reasoning trials in which a conclusion might be reached by just checking the truth of the concluding statement. Since all 25 valid syllogisms resulted in true conclusions and 20% of the 25 invalid syllogisms also resulted in true conclusions, if the response was solely based on the truth of the conclusion, error rate should be systematically larger for invalid syllogisms compared to valid which was not the case. Thus, the behavioral results provide evidence against the hypothesis that participants used this strategy (heuristic model) in deductive reasoning trials.

Paradoxes require deep semantic comprehension and mapping procedures, like context and world knowledge integration, reanalyzing meanings, and semantic analysis beyond the surface meaning of the words within, even if they have traditionally simple syntactic structure. These facts could explain the increase in reaction time for paradoxical compared to deductive reasoning. Our results, also, showed that the group mean response accuracy for paradoxical trials was approximately 50%. That was the expected outcome since in paradoxes, there is no right or wrong response. The meaning of an erroneous response in paradoxes is relative and it depends on each subject’s critical point of view.

### The Neural Substrate of Paradoxes

This is, to our knowledge, the first study to specifically investigate the neural substrate of paradoxical reasoning, using fMRI. Paradoxes activated a fronto-temporal brain network, including bilateral inferior frontal gyrus (BA 45/47), middle temporal gyrus (BA 21/19/37), left medial frontal gyrus (BA 8), and superior frontal gyrus (BA 9). A previous study that has used the same design as the current one was an ERP electrophysiological study (Papageorgiou et al., 2016). Their results suggested that deductive reasoning activated parieto-occipital areas associated with attention and subsequent memory processing, whereas paradoxical reasoning engaged frontal attention mechanisms. Both studies suggest the involvement of different neural networks for deductive and paradoxical reasoning, emphasizing the specific involvement of parietal cortical areas for the former and frontal cortical areas for the latter.

Since this is the first fMRI study attempting to investigate the specific neural network that is engaged in paradoxical reasoning, it is interesting to discuss the role of these areas in other cognitive tasks as suggested by the relevant literature. The left middle temporal gyrus is important for language comprehension and more specifically for lexical semantic processing at the sentence level and in semantic integration of word meaning in the sentence context (Vigneau et al., 2006; Chow et al., 2008; Bosco et al., 2017). Furthermore, this brain area has a key role within the language network, since it has multitudinous connections with different cortical association areas (Rapp et al., 2012). Moreover, right temporal lobe seems to be activated when there is increased need for lexical semantic integration and retrieval (Zempleni et al., 2007).

The inferior frontal gyrus (BA 45/47) plays a key role in semantic language comprehension at the sentence level (Petrides, 2005). The left inferior frontal gyrus is a key region of this fronto-temporal network (especially BA 45/47) and has been related with several higher order control processes, which regulate the selection among multiple competing responses (Petrides, 2005). This brain region, also, plays an important part in semantic retrieval on a sentence level (Petrides, 2005; Sakai, 2005). Moreover, recent studies propose that inferior frontal gyrus (BA 45/47) seems to have a crucial role in semantic and pragmatic processing, including pragmatic and semantic violations. The right inferior frontal gyrus is, also, involved when language processing demand increases (Zempleni et al., 2007).

We could thus hypothesize that this fronto-temporal network plays a dominant role in paradoxical syllogism, as this network is engaged in the comprehension of other non-literal expressions, such as metaphors (Rapp et al., 2004; Mashal et al., 2008; Bohrn et al., 2012), idioms (Zempleni et al., 2007; Bohrn et al., 2012), irony (Shibata et al., 2009; Bohrn et al., 2012), and metonymy resolution (Rapp et al., 2011). The activation of this network in our study might suggest the integration of semantic and world knowledge during comprehension of paradoxes.

Another possible interpretation of these results could be based on the hypothesis that paradoxical syllogisms engage a process of belief revision (retracting a proposition that makes a belief system incoherent by choosing to withdraw the proposition that implies a minimal cost). The Achilles paradox leads to a conflict between prior beliefs—the fleet-footed Achilles will manage to pass the slow-moving tortoise—and logic—Achilles will never surpass the tortoise, since there is an infinite number of points that Achilles must reach, before surpassing it, and by that time the tortoise will have already gone. Thus, the subject is obliged to choose the one proposition among these two that implies minimal cost. Goel et al. (2000) proposed the activation of right prefrontal cortex during belief-logic conflict arguments which is in line with the current study, activation results during paradoxical reasoning.

Finally paradoxical reasoning might be close to enthymematic reasoning, that is reasoning where a premise is missing (thus blocking inference) and an additional premise must be added to the initial stock of premises to carry out the inference process (see for example Brun and Rott, 2013). Prado et al. (2020) tried to investigate the neural substrates of argumentative reasoning. Namely, they designed a task, in which participants had to read and evaluate the same statement. This statement was presented either as an assertion or as the conclusion of an argument (enthymematic categorical syllogisms). They found that there was greater activation of the medial prefrontal cortex in conclusions of arguments than assertions, suggesting that argumentative reasoning might depend on mechanisms which support meta-representational processes.

### The Neural Substrate of Deductive Reasoning

In previous studies investigating the neural substrate of reasoning, many tasks were used that differed in the type of deductive arguments (relational, conditional, probabilistic, and inductive problems), while only rarely investigators have used a comparison with a non-logical task (Knauff et al., 2002). Our findings indicate that both valid and invalid deductive reasoning engages a neural network comprising bilateral precuneus (BA 7/19), inferior parietal lobule (BA 40) and right middle frontal gyrus (BA 46/10), left inferior frontal gyrus (BA 44), right inferior frontal gyrus (BA 9), left lingual gyrus (BA 17), cingulate gyrus (BA 32), precentral gyrus (BA 6), and right inferior occipital gyrus (BA 17).

Our results are consistent with previous neuroimaging studies, which favor the idea that deductive reasoning is supported by a distributed network of regions including frontal and parietal areas (Knauff et al., 2002; Goel and Dolan, 2004; Monti et al., 2007; Reverberi et al., 2012). Our results are, also, consistent with the idea that reasoning consists complex cognitive process that depends on the activation of several brain areas, including frontal and parietal cortices. This function is, also, linked to working memory, attention and language operations. Consequently, our neuroimaging results provide further support, based on the dual-processing hypothesis in reasoning, that our participants used the analytic slow, sequential process for making logical inferences activating parieto-occipital brain areas and working memory associated areas (De Neys, 2006; Stephens et al., 2020).

## Conclusion

In conclusion, the present study demonstrated that two different types of argument, paradoxical and deductive reasoning, might rely on distinct neural substrates. Deductive reasoning mainly engaged fronto-parietal brain activation patterns, whereas paradoxes mainly engaged fronto-temporal brain activation patterns. These findings propose that reasoning might not rely on a unitary brain network, suggesting that different mechanisms are engaged in different reasoning processes. Our study provides a basis for future studies on human physiological as well as pathological reasoning and enabling us to investigate different aspects of reasoning.

## Data Availability Statement

The raw data supporting the conclusions of this article will be made available by the authors, without undue reservation.

## Ethics Statement

The studies involving human participants were reviewed and approved by Ethics Committee of Eginition University Hospital. The patients/participants provided their written informed consent to participate in this study.

## Author Contributions

AB performed the data collection and participated in study design, data analysis, and writing of the manuscript. CP conceived the original idea and participated in study design and writing of the manuscript. EK participated in data analysis and writing of the manuscript. ET participated in study design and writing of the manuscript. CK participated in data analysis and editing of the manuscript. NS had the overall responsibility for the project and participated in study design, data analysis, and writing and editing of the manuscript. All authors contributed to the article and approved the submitted version.

## Funding

The study was funded by the Regional Governor of Attica.

## Conflict of Interest

The authors declare that the research was conducted in the absence of any commercial or financial relationships that could be construed as a potential conflict of interest.

## Publisher’s Note

All claims expressed in this article are solely those of the authors and do not necessarily represent those of their affiliated organizations, or those of the publisher, the editors and the reviewers. Any product that may be evaluated in this article, or claim that may be made by its manufacturer, is not guaranteed or endorsed by the publisher.
