# Supplementary material for: Paradoxical Reasoning: An fMRI Study
Source: Front Psychol. 2022 May 2;13:850491. doi: 10.3389/fpsyg.2022.850491 (PMC9113220; doi:10.3389/fpsyg.2022.850491)
Supplement: Supplementary file 1 [file Data_Sheet_1.DOCX]

**Paradoxical Reasoning: an fMRI Study**

**SUPPLEMENTARY MATERIAL: The reasoning trials used**

|  | **Reasoning Trials** |  | **Paradoxes** |
| --- | --- | --- | --- |
| **1** | All youngsters are animals.  Some ravens are youngsters.  Some ravens are animals. | **1** | Achilles and the tortoise decides to race. The tortoise gains a head start, because it runs slowly. Therefore, the tortoise will always be ahead of Achilles |
| **2** | No jug in this cupboard is new.  All jugs in this cupboard are cracked.  Some cracked things in this cupboard are not new. | **2** | An object is at rest when it occupies a space equal to itself. A moving object occupies a place of its own size at every moment of its trajectory. Therefore, at every moment of its trajectory a moving arrow is at rest, immovable. |
| **3** | Some cats have no tails.  All cats are mammals.  Some mammals have no tails. | **3** | A Cretan claims that all Cretans are liars. |
| **4** | No tree is edible.  Some trees are green.  Some green things are not edible. | **4** | A male barber shaves every man in town who does not shave himself but no one else. The barber shaves himself? |
| **5** | All apples of my garden are nutritious.  All nutritious fruits are ripe.  Some ripe fruits in my garden are apples. | **5** | If a hotel with an infinite number of rooms is full, it can still take in more guests. |
| **6** | All coloured flowers are fragrant.  No fragrant flower grows indoors.  No flower which grows indoors is coloured. | **6** | If there is an exception to every rule, then every rule must have at least one exception. The exception to this one being that it has no exception. |
| **7** | Some small birds eat honey.  All birds which eat honey are coloured.  Some coloured birds are small. | **7** | Several documents include pages which have the phrase: ‘This page was intentionally left blank’. Is that page blank? |
| **8** | No human is perfect.  All perfect creatures are mythical.  Some mythical creatures are not human. | **8** | "Moderation in all things, including moderation". |
| **9** | Incompetent men are men who always make mistakes.  Some men who always make mistakes work here.  Some men who work here are incompetent. | **9** | The first number which would be regarded as more boring than interesting would get interesting due to this particular fact. |
| **10** | No attack on civilians is a justifiable military operation.  Some military operations were attacks on civilians.  Some military operations were not justifiable. | **10** | A choice between two outcomes of exactly the same value can be a rational choice? |
| **11** | All virtuous men have good judgment.  Some of my roommate’s friends lack good judgment.  Some of my roommate’s friends are not virtuous men. | **11** | Will anything happen if an unstoppable force strikes an immovable object? |
| **12** | All virtuous men possess the virtue of moderation.  No one who gets drunk deliberately possesses the virtue of moderation.  No one who gets drunk deliberately is a virtuous person. | **12** | The night sky is dark if there exists an infinity of stars, covering every part of the celestial sphere. |
| **13** | All Martians are inhabitants of other planets.  Some Martians are not intelligent.  Some intelligent creatures are not inhabitants of other planets. | **13** | The existence of evil seems to be incompatible with the existence of an omnipotent, omniscient, and morally perfect God. |
| **14** | All men are capable of reasoning.  No non-human creature is capable of reasoning.  No non-human creature is human. | **14** | If all truths are recognisable, then all truths must in fact be known. |
| **15** | Whoever corrupts the youth of his city harms himself.  Socrates does not harm himself.  Socrates does not corrupt the youth of his city. | **15** | If truth does not exist, then the phrase “The truth does not exist” is a truth and therefore proves itself wrong. |
| **16** | No herbivore has sharp teeth  No cow has sharp teeth.  All cows are herbivores. | **16** | Can an omnipotent being make a rock so heavy that even he cannot lift? |
| **17** | No man who commits a crime unintentionally would have been prevented by the possibility of punishment.  Some murderers commit crimes unintentionally.  Some murderers will not be prevented by the possibility of punishment. | **17** | When one pursues happiness itself, one is miserable; but, when one pursues something else, one achieves happiness. |
| **18** | All poisons are bitter.  Arsenic is not bitter.  Arsenic is not poison. | **18** | Water is more useful than diamonds, yet is a lot cheaper. |
| **19** | All men who eat meat cannot declare themselves against hunting.  You eat meat.  Therefore, you cannot declare yourself against hunting. | **19** | Increasing the price of bread makes poor people eat more of it. |
| **20** | All mathematicians are clever.  Some youths are mathematicians.  Some youths are clever. | **20** | Increases in production efficiency lead to even larger increases in demand. |
| **21** | No fish is a mammal.  Some creatures which live in the water are mammals.  Some creatures which live in the water are not fish. | **21** | What is better than eternal bliss? Nothing. But a slice of bread is better than nothing. So a slice of bread is better than eternal bliss. |
| **22** | All cats are furry mammals.  Some pets are not furry mammals.  Some pets are not cats. | **22** | Can a person drown in the source of eternal life? |
| **23** | All the first degree murder is premeditated (deliberate/wilful) homicides.  No premeditated (deliberate/wilful) homicide is not an act of self-defence.  No act of self-defence is not first degree murder. | **23** | Did you know that “0.999…” equals “1”? |
| **24** | Some evergreens are subject of worship.  All evergreen are trees.  Some trees are subjects of worship. | **24** | A greedy man gives away his cash with sorrow. However, he doesn't have cash with sorrow, so he gives away what he doesn't have. |
| **25** | Some snakes are not venomous.  All snakes are reptiles.  Some reptiles are not venomous. | **25** | People can make decisions based not on what they actually want to do, but on what they think that other people want to do. Hence everyone decides to do something no one actually wants to do and does what he believes that everyone else wants to do. |
| **26** | No snakes are mammal.  Some mammals are aquatic.  Some aquatic creatures are not snakes. |  |  |
| **27** | All ants are insects.  Some ants have wings.  Some winged creatures are insects. |  |  |
| **28** | Some predatory birds are eagles.  All eagles have exceptional sight.  Some creatures with exceptional sight are predatory birds. |  |  |
| **29** | All men are mortal.  All Greeks are men.  All Greeks are mortal. |  |  |
| **30** | No reptiles have fur.  All snakes are reptiles.  No snakes have fur. |  |  |
| **31** | All kittens are playful.  Some pets are kittens.  Some pets are playful. |  |  |
| **32** | No homework is fun.  Some reading is homework.  Some reading is not fun. |  |  |
| **33** | No healthy food is fattening.  All cakes are fattening.  No cake is healthy. |  |  |
| **34** | All horses have hooves.  No humans have hooves.  No humans are horses. |  |  |
| **35** | No slothful person succeeds in examinations.  Some pupils succeed in examinations.  Some pupils are not slothful. |  |  |
| **36** | All informative things are useful.  Some websites are not useful.  Some websites are not informative. |  |  |
| **37** | All fruit is nutritious.  All fruit is tasty.  Some tasty things nutritious. |  |  |
| **38** | Some cups are beautiful.  All cups are useful.  Some useful things are beautiful. |  |  |
| **39** | All industrious boys in this school have red hair.  Some industrious boys in this school are boarders.  Some boarders in this school have red hair. |  |  |
| **40** | All the planets are illuminated by the sun.  Venus is a planet.  Venus is illuminated by the sun. |  |  |
| **41** | All arithmetic operations are useful.  Addition is an arithmetic operation.  The addition is useful. |  |  |
| **42** | Some children are naughty.  Some children are boys.  Some boys are naughty. |  |  |
| **43** | All the hens are birds.  Some birds are flying in the sky.  All the hens fly in the sky. |  |  |
| **44** | All women are teachers.  Some teachers are strict.  All women are strict. |  |  |
| **45** | All French people are lazy.  Some French people are bookish.  All bookish people are lazy. |  |  |
| **46** | No lawyer is a man.  No actor is a lawyer.  Some actors are not men. |  |  |
| **47** | All ladies are polite.  All ladies are women.  No woman is polite. |  |  |
| **48** | Some men are teachers.  Some actors are men.  No actor is a teacher. |  |  |
| **49** | Some students are boys.  Some students are girls.  All boys are girls. |  |  |
| **50** | Some mollusks are invertebrates.  Some urticarial are invertebrates.  All mollusks are urticarial. |  |  |
